# Exogenous 24-epibrassinolide ameliorates tolerance to high-temperature by adjusting the biosynthesis of pigments, enzymatic, non-enzymatic antioxidants, and diosgenin content in fenugreek

**DOI:** 10.1038/s41598-023-33913-6

**Published:** 2023-04-24

**Authors:** Shahla Sheikhi, Amin Ebrahimi, Parviz Heidari, Mohamad Reza Amerian, Sajad Rashidi-Monfared, Hadi Alipour

**Affiliations:** 1 Department of Agriculture and Plant Breeding, Faculty of Agriculture, Shahrood University of Technology, Semnan, Iran; 2 Plant Breeding and Biotechnology Department, Faculty of Agriculture, Tarbiat Modares University, Tehran, Iran; 3 Department of Plant Production and Genetics, Faculty of Agriculture, Urmia University, Urmia, Iran

**Keywords:** Physiology, Plant sciences

## Abstract

High-temperature stress is widely considered a main plant-growth-limiting factor. The positive effects of 24-epibrassinolide (EBR) as analogs of brassinosteroids (BRs) in modulating abiotic stresses have led this hormone to be referred to as a growth regulator in plants. The current study highlights the influence of EBR on enhancing tolerance to high-temperature and altering the diosgenin content in fenugreek. Different amounts of EBR (4, 8, and 16 μM), harvesting times (6, and 24 h), as well as temperature regimes (23 °C, and 42 °C) were, used as treatments. EBR application under normal temperature and high-temperature stress resulted in decreased malondialdehyde content and electrolyte leakage percentage, while the activity of antioxidant enzymes improved significantly. Exogenous EBR application possibly contributes to activating the nitric oxide, H_2_O_2_, and ABA-dependent pathways, enhancing the biosynthesis of abscisic acid and auxin, and regulating the signal transduction pathways, which raises fenugreek tolerance to high-temperature. The *SQS* (eightfold), *SEP* (2.8-fold), *CAS* (11-fold), *SMT* (17-fold), and *SQS* (sixfold) expression, considerably increased following EBR application (8 μM) compared to the control. Compared to the control, when the short-term (6 h) high-temperature stress was accompanied by EBR (8 μM), a sixfold increase in diosgenin content was achieved. Our findings highlight the potential role of exogenous 24-epibrassinolide in mitigating the high-temperature stress in fenugreek by stimulating the biosynthesis processes of enzymatic and non-enzymatic antioxidants, chlorophylls, and diosgenin. In conclusion, the current results could be of utmost importance in breeding or biotechnology-based programs of fenugreek and also in the researches related to the engineering of the biosynthesis pathway of diosgenin in this valuable plant.

## Introduction

*Trigonella foenum-graecum* is a dicotyledonous plant in the *Fabaceae* family, the subfamily of *Papilionaceae,* which is used from time immemorial. It is currently used for medicinal purposes, as a vegetable in the human diet^1^, and also in phytoremediation of polluted soils^1^. Major bioactive phytochemicals constituents of fenugreek are classified into alkaloids, amino acids, coumarins, flavonoids, and saponins^2^. Fenugreek has one type of steroidal saponins known as diosgenin, which was used for cardiovascular diseases, cancer, and anti-aging treatments^3^. This plant grows natively in almost all parts of Iran and is cultivated in most provinces^4^. Early cultivation of fenugreek in Iran leads to spring frosts; while in late cultivation, the plant is exposed to high-temperatures, especially at the beginning of flowering. There is not any breeding background for this crop in Iran and local landraces are commonly used by farmers^4^. Due to the special geographical and climatic conditions of Iran, the occurrence of environmental stresses would be quite inevitable, so the identification of stress-tolerant plants with high yield potential or the use of various stimulants to improve plants tolerance, especially medicinal plants, seems necessary^5^.

Among the environmental stresses, global warming and the increased temperature have detrimental effects on plant growth and yield and significantly reduce soil productivity^6^. Disruption of photosynthesis function, denaturation of proteins and generation of heat shock proteins, decomposition of enzymes, reactive oxygen species accumulation, and disruption of plant cell walls are primary and vital damages caused by the high-temperature stress^7^. In previous studies, the adverse effects of high-temperature stress on the quality and quantity of fenugreek were well established, although biotic and abiotic stresses have effectively increased the content of some important secondary metabolites, such as diosgenin and trigonelline in fenugreek^5,8–11^.

Recently, many researchers focused on classical methods of plant breeding and biotechnology to enhance the plant's tolerance to a wide range of environmental stresses^5,8,9^. Although quite expensive, complicated, and time-consuming, screening, and identifying the superior genotypes could be listed as a crucial step in breeding programs^12^.When this key step is carried out in the same climatic region in which the domestic cultivars are used, more satisfactory results are guaranteed. Even though the wild genotypes may have unacceptable yield, they could irreplaceably be useful when enhancing the plants's nutritional quality or their tolerance to environmental stresses concerned^13^. Recently, the exogenous application of different elicitors such as osmoprotectants^14,15^ phytohormones^16^, polyamines^17,18^, cold plasma^10^, and melatonin^11^ have been reported to reduce the damaging effects of stress on plants. Phytohormones, the plant growth regulators, have vital roles in stress adaptations^19^. Around 40 years ago, a new period began in the researches concerning plant growth regulators with the discovery of a new phytohormone, brassinosteroids (BRs)^19^. BRs undeniably impact on plants's growth and development and play numerous roles in physiological functions, including seed germination, cell division, and elongation, floral organ elongation, pollen tube development, xylem differentiation, vascular-tissue differentiation, biosynthesis of ethylene, senescence, root development, and photosynthesis^19–22^. Furthermore, the BRs could increase stress tolerance and acclimation by changing genes expression^7^. Previous researches confirmed that the BRs could adjust environmental stresses condition independently or via crosstalk with other phytohormones such as abscisic acid (ABA)^22,23^. The BRs signal transduction is based on phosphorylation/dephosphorylation between membrane steroid receptors, triggering a signal cascade in the cytoplasm and activation of downstream transcription factors. BES1/BZR1 is a critical transcription factor in the BR signaling that could enter the nucleus and bind to the BRs responsive gene promoter (REF). Under abiotic stress, the accumulation of the BZR1 in the nucleus increases, and causes the genes expression related to cell growth^22^.

The content of secondary metabolites could be increased by the various stimulants, including abiotic stresses, radiation (ultraviolet, gamma, etc.) and different hormones (salicylic acid, jasmonates, BRs). The efficiency of these elicitors on the content of secondary metabolites, essential oil components and other physiological, and morphological properties have previously been examined in medicinal plants^10,24^. The effects of environmental stresses on medicinal plants would be somewhat different from other plants, especially crops. It should be well noted that although abiotic stress in medicinal plants leads to a decrease in yield, ultimately the content of secondary metabolites as the most valuable medicinal compounds increases^25^. Therefore, the application of abiotic stresses in medicinal plants according to the purpose of their use (vegetative tissue or secondary metabolism) requires special expertise, particularly in identifying the intensity of this stress and the plant growth stage as well as using various stimulants. The concentration of stimulus and duration of exposure may highly contribute to increasing the performance of the desired stress and stimulus. These points are crucial as applying inappropriate stress intensity, incorrect growth stage, and non-optimal elicitor concentration could lead to a severe decline in yield and even in secondary metabolites content^11,26^.

The biochemical and physiological responses of Iranian fenugreek native cultivars to high-temperature stress has remained still unknown. Accordingly, the current study was carried out aimed at assessing the molecular, physiological, and biochemical responses of native Boshruyeh ecotype to high-temperature. We tried to highlight the capability of 24-epibrassinolide to make the plant more tolerant to high-temperature stress and to increase the diosgenin content in fenugreek as well.

## Results

### Chlorophyll and carotenoid contents

**Figure 1:**
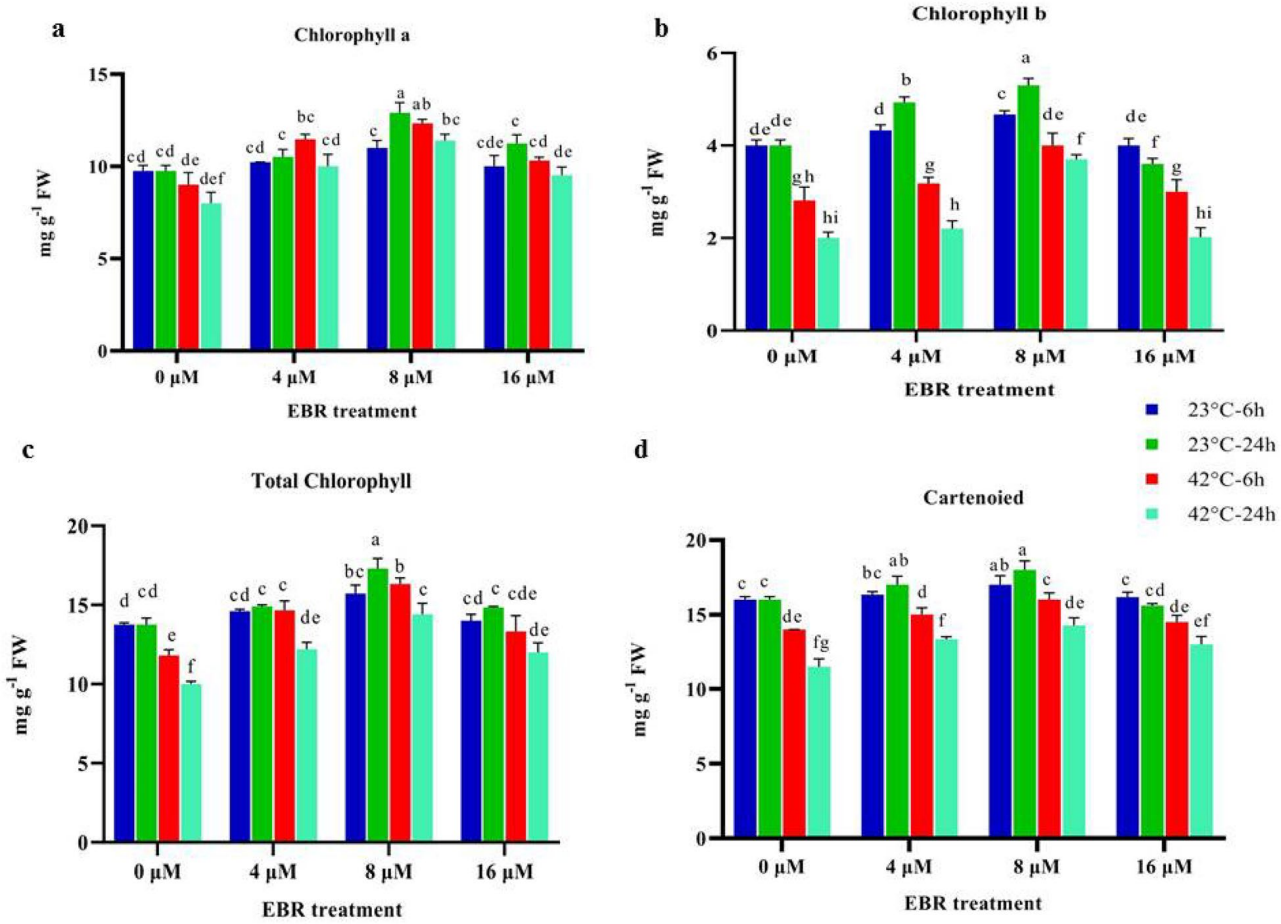
The effects of temperature, harvesting times, and different levels of EBR on chlorophyll a (**a**), chlorophyll b (**b**), total chlorophyll (**c**), and carotenoid (**d**). Duncan method at a 1% probability level was applied to compare mean values. The columns having similar letters had no significant difference.

The results indicated that chlorophyll a, b and, total chlorophyll content were significantly decreased by (*p* < 0.01) 20%, 55%, and 35% under high-temperature stress (42 °C, 24 h after the onset of the high-temperature stress) compared to the control (23 °C, non-application of EBR). Chlorophyll a, b, total chlorophyll, and carotenoid contents were remarkably enhanced by 31%, 30%, 30%, and 15% with EBR application (8 μM, 24 h) under normal temperature condition (23 °C) compared to the non-application of EBR, respectively. Also, the pigment contents were significantly increased with EBR application at all levels under high-temperature compared to the non-application of EBR. For instance, chlorophyll a, chlorophyll b, and total chlorophyll content was significantly (*p* < 0.01) rose by 42%, 90%, and 47% with EBR application (8 μM) under high-temperature (24 h) compared to the non-application of EBR (under high-temperature) (Fig. 1).

The results revealed that the content of carotenoid decreased significantly at 6 and 24 h during high-temperature stress compared to the control. Carotenoid content increased in response to EBR application at all levels under high-temperature in comparison with the untreated samples (Fig. 1). In addition, the application of EBR (8 μM) under high-temperature not only significantly prevented the reduction in the content of chlorophyll and carotenoid, but also enhanced these pigments concerning the high-temperature condition (without EBR application). Carotenoid content significantly rose (*p* < 0.01) by 15% with the EBR application (8 μM) under high-temperature (24 h) compared to the non-application of EBR (under high-temperature) (Fig. 1).

### Indicators related to cell membrane vulnerability

**Figure 2:**
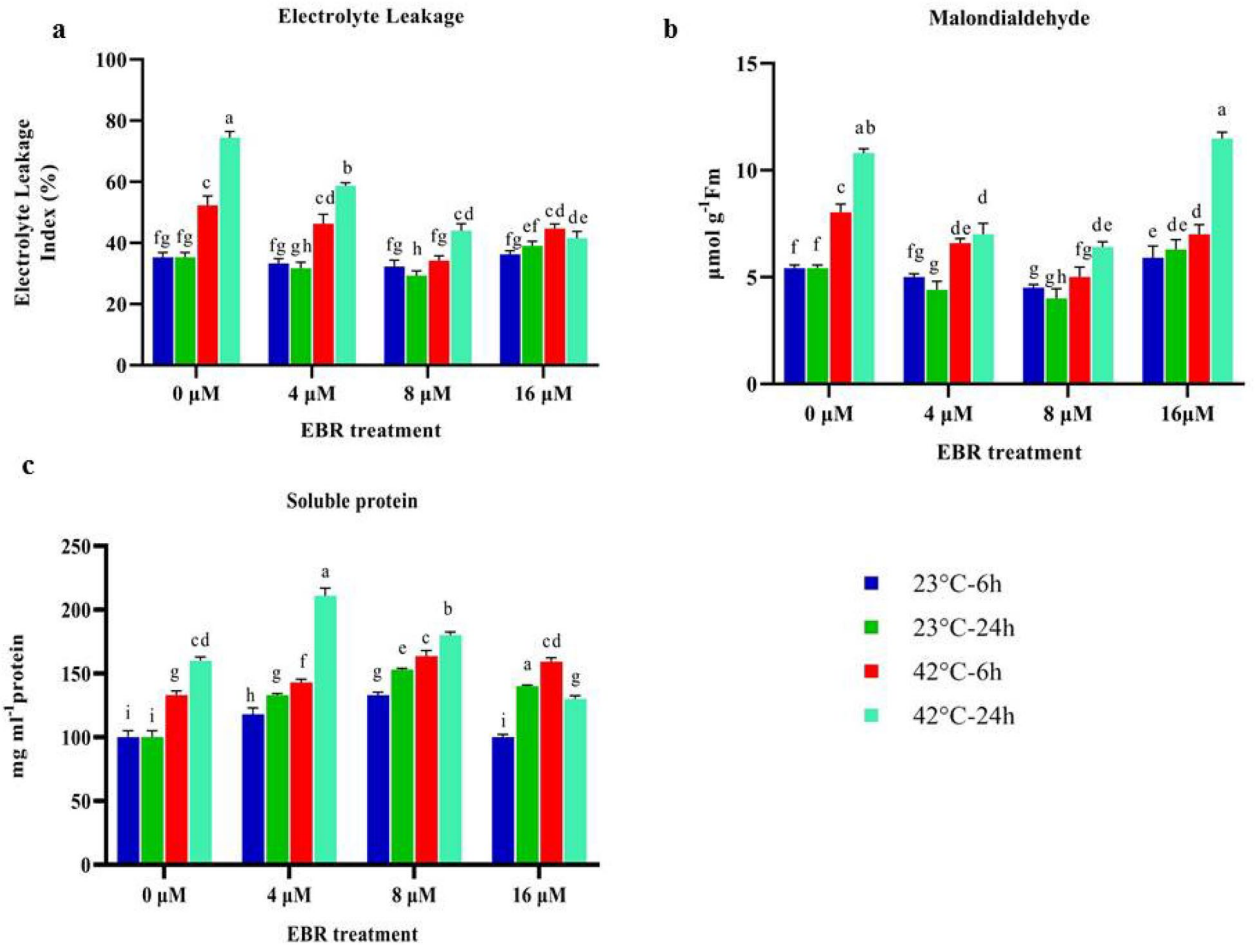
The effects of temperature, harvesting times, and different levels of EBR on electrolyte leakage (**a**), MDA (**b**), and total soluble protein (**c**). Duncan method at a 1% probability level was applied to compare mean values. The columns having similar letters had no significant difference.

The electrolyte leakage percentage and malondialdehyde content (MDA) significantly increased (*p* < 0.01) by 43% and 45% after 6 h during high-temperature stress compared to normal temperature (23 °C), and they reached their highest content at 24 h. MDA content and electrolyte leakage index in sprayed plants grown under normal temperature condition (23 °C) were significantly reduced (*p* < 0.01) by 29% and 23% compared to plants not treated with EBR, respectively (Fig. 2).

The application of different levels of EBR (under high-temperature) significantly reduced the percentage of electrolyte leakage as well as MDA content in relation to the non-EBR application. The lowest amount of these two traits was observed applying 8 μM of EBR at 6 h (25.33% and 5 µmol g^−1^ FW) under high-temperature stress, indicating that the hormone application combined with high-temperature stress effectively prevented electrolyte leakage percentage and MDA content from increasing (Fig. 2).

### Antioxidant enzymes, H_2_O_2,_ and nitric oxide content

The results of present study showed that the total soluble protein content increased by 38% and 63% during the high-temperature stress at 6 and 24 h compared to the control (23 °C), respectively. Total soluble protein content was significantly elevated at 6 and 24 h (during the high-temperature stress) along with 4 μM of EBR and was significantly different from other treatments (Fig. 2).

**Figure 3:**
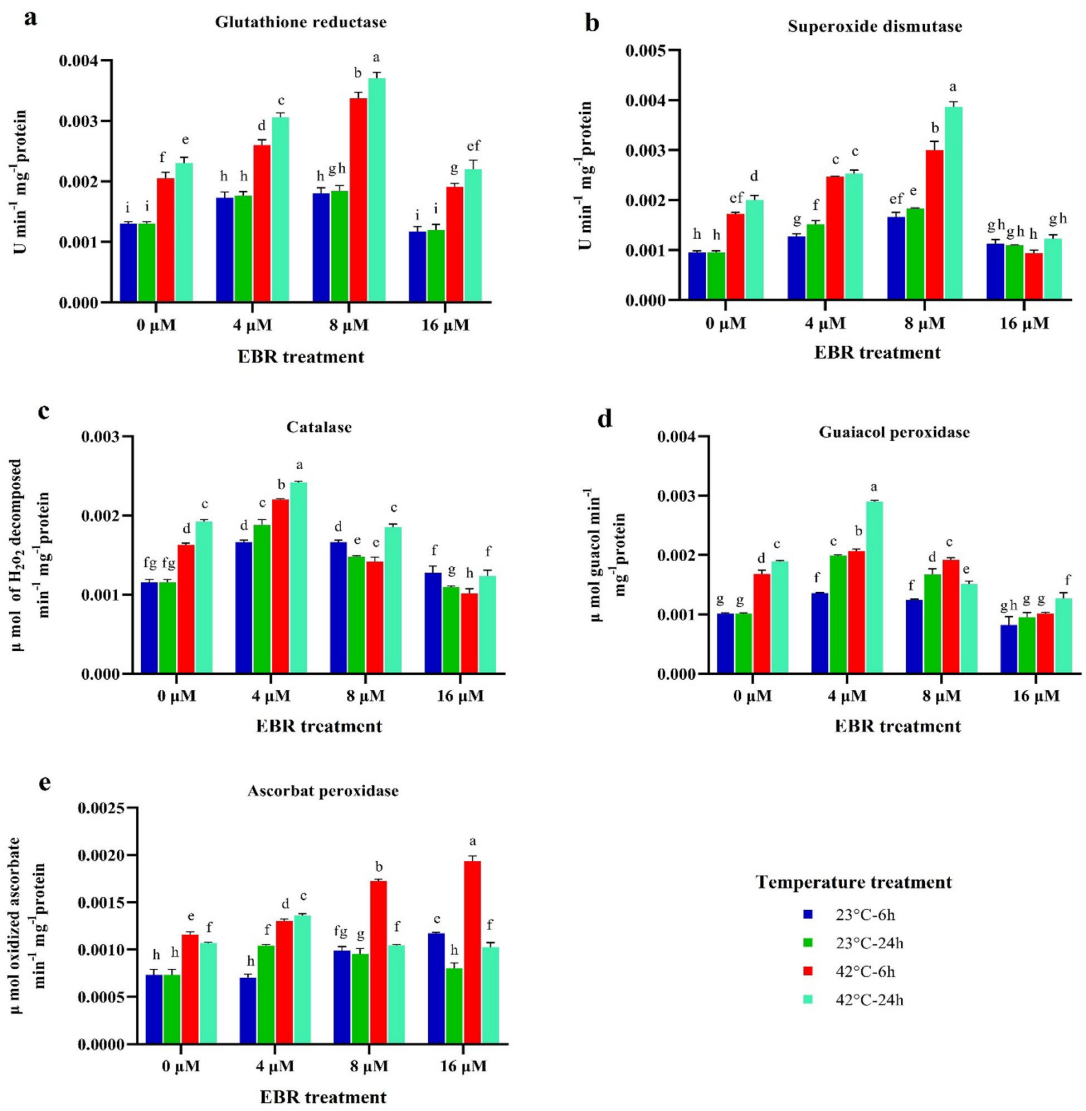
The effects of temperature, harvesting times, and different levels of EBR on superoxide dismutase (**a**), glutathione reductase (**b**), catalase (**c**), guaiacol peroxidase (**d**) and ascorbate peroxidase (**e**). Duncan method at a 1% probability level was applied to compare mean values. The columns having similar letters had no significant difference.

The activity of glutathione reductase (GR) and superoxide dismutase (SOD) varied similarly; indicating the activity of both enzymes increased by high-temperature stress and had a significant difference with normal temperature. This upward trend continued in both enzymes with the application of 4 and 8 μM of EBR so that their highest activity was observed in the 8 μM of EBR and 24 h (0.0387 and 0.0377 U min^−1^ mg^−1^ protein, respectively) (Fig. 3) under high-temperature stress. The results proved that in comparison with the control (23 °C), 6 and 24 h of the temperature stress resulted in the catalase activity (CAT) being significantly increased. The activity of this crucial enzyme underwent a significant rise after 6 and 24 h in EBR-treated samples. The highest activity of catalase was 0.0024 μmol of H_2_O_2_ decomposed min^−1^ mg^−1^ protein in the treatment of 4 μM of EBR at 24 h, indicating the significant role of this enzyme in raising the plant tolerance to stress (Fig. 3).

The activity of guaiacol peroxidase (GP_X_) significantly increased (*p* < 0.01) by 80% and 100% at 6 and 24 h during high-temperature stress compared to the control (23 °C), respectively. Under the high-temperature stress along with 4 and 8 μM concentrations of EBR, its activity rose in comparison with the non-application of EBR, although this difference was not statistically significant. The highest activity of GP_X_ was 0.0028 μmol guaiacol min^−1^ mg^−1^ protein applying 4 μM of EBR (24 h), which significantly differed from the other treatments (Fig. 3). The ascorbate peroxidase (APX) activity significantly rose following the high-temperature stress (6 h and 24 h) compared to the control (23 °C). Its activity was significantly elevated in the combined treatment, i.e. EBR application and the high-temperature stress, reaching the highest level (0.0018 μmol oxidized ascorbate min^−1^ mg^−1^ protein) at 6 h applying 16 μM of EBR (Fig. 3).

In total, spraying EBR (8 μM) on plants grown under normal temperature condition significantly (*p* < 0.01) increased the activity of GR, SOD, CAT, GP_X_, and APX by 27, 25, 15, 35, and 24% compared to plants not treated with EBR, which shows the effect of this hormone in condition of normal temperature and high-temperature stress (Fig. 3).

**Figure 4:**
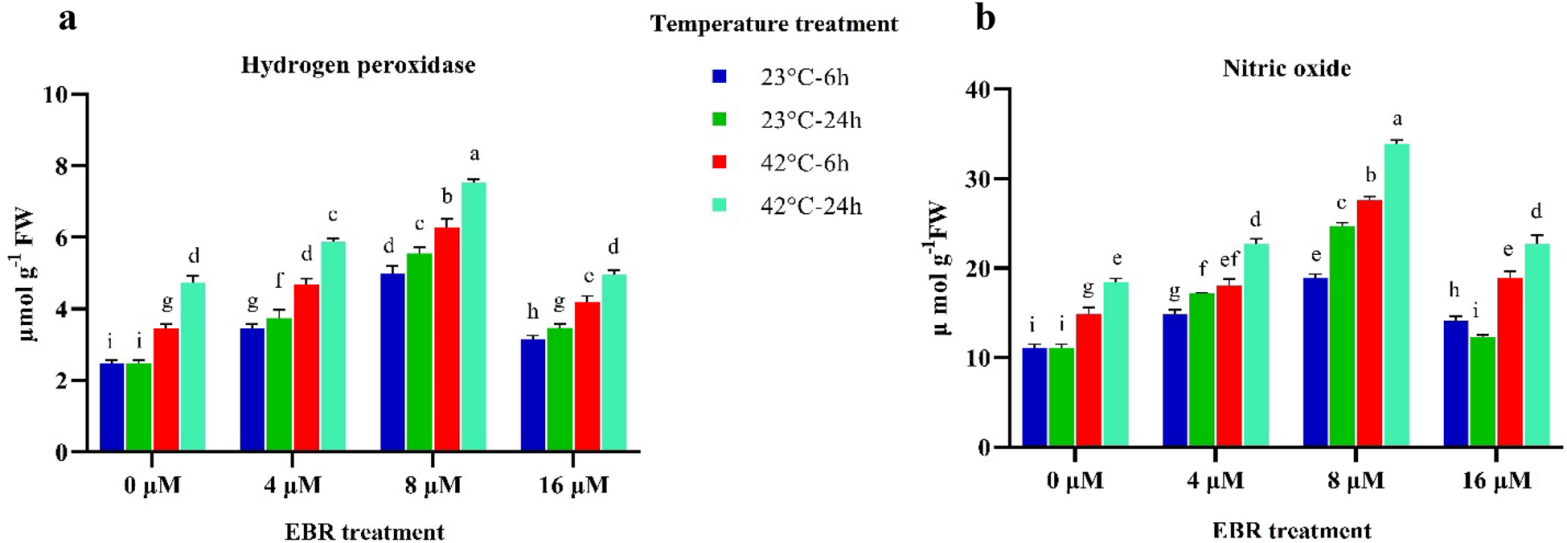
The effects of temperature, harvesting times, and different levels of EBR on H_2_O_2_ (**a**), and NO (**b**). Duncan method at a 1% probability level was applied to compare mean values. The columns having similar letters had no significant difference.

Applying different levels of EBR substantially raised the amount of nitric oxide and hydrogen peroxide (Fig. 4). The amount of nitric oxide and hydrogen peroxide following the application of EBR (8 μM) in plants grown under normal condition improved by 30 and 55%, respectively, compared to the absence of this hormone.High-temperature stress and more effectively, EBR application significantly increased nitric oxide and hydrogen peroxide contents. These two compounds both rose more when applying EBR was accompanied by high-temperature stress. The highest content of nitric oxide (35 µmol g^−1^ FW) and hydrogen peroxide (7.4 µmol g^−1^ FW) was observed under high-temperature (24 h) along with the EBR application (8 μM) (Fig. 4).

### Abscisic acid (ABA), and auxin content

**Figure 5:**
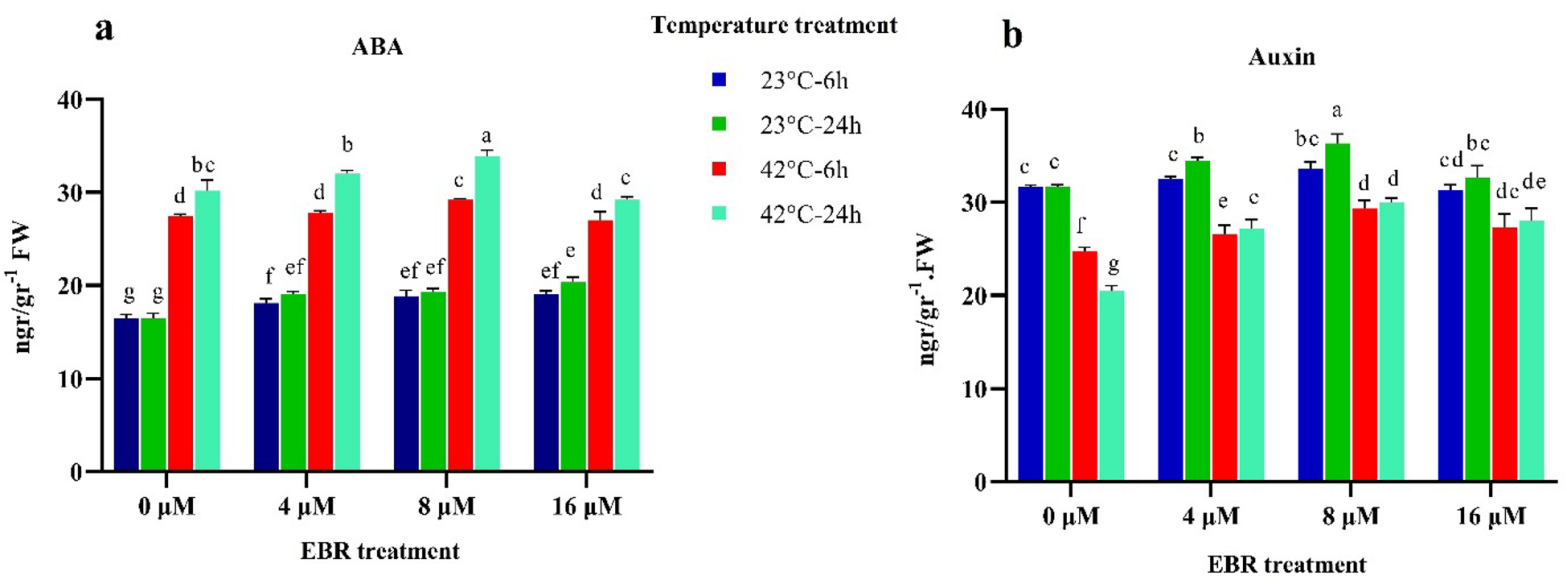
The effects of temperature, harvesting times, and different levels of EBR on Abscisic acid (**a**) and Auxin content (**b**). Duncan method at a 1% probability level was applied to compare mean values. The columns having similar letters had no significant difference.

As shown in Fig. 5, high-temperature stress at 24 h raised the content of abscisic acid (50%) and reduced the content of auxin (40%) compared to the control (23 °C). In the control, the content of these two hormones increased significantly (*p* < 0.01) with the use of different levels of EBR to the level of 8 μM, but decreased by applying 16 μM of EBR. Exogenous application of different levels of EBR during stress enhanced the content of endogenous abscisic acid (Fig. 5). The highest content of abscisic acid was 33 ng g^−1^ FW applying 8 μM of EBR at 24 h under high-temperature stress. Besides, the endogenous content of auxin rose significantly after the use of EBR. (Fig. 5). Auxin content increased by 40% in response to EBR application (8 μM) under high-temperature at 24 h in comparison with the untreated samples (Fig. 5).

### Gene expression profiles, and diosgenin content

**Figure 6:**
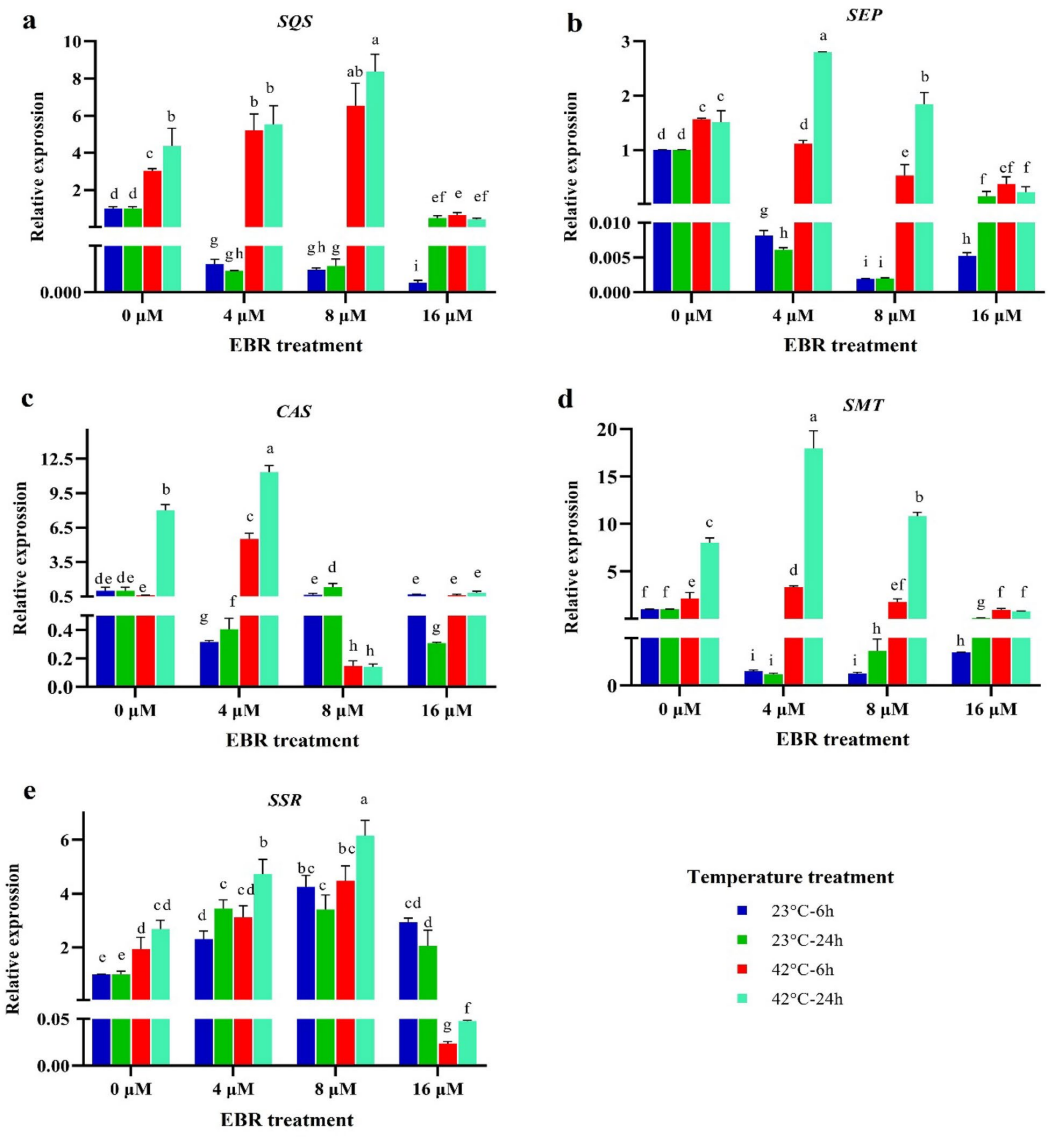
The effects of temperature, harvesting times, and different levels of EBR on the expression levels of *SQS* (**a**)*, SEP* (**b**)*, CAS* (**c**), *SMT* (**d**), and *SSR* (**e**). Duncan method at a 1% probability level was applied to compare mean values. The columns having similar letters had no significant difference.

The squalene synthase (*SQS*) gene expression was significantly reduced under normal temperature (23 °C), 6 and 24 h after using different concentrations of EBR compared to the control (23 °C without EBR). Under high-temperature stress at both 6 and 24 h without EBR treatment, the expression of this gene rose by 2.7-fold and fourfold in relation to the control, respectively (Fig. 6). Besides, the squalene epoxidase (*SEP*) expression significantly lowered under the control (23 °C) after using different levels of EBR, in contrast, its expression under high-temperature stress without EBR the application rose compared to the control. The *SEP* expression increased by 2.8-fold after the onset of the high-temperature stress along with the application of EBR (4 μM, at 24 h) (Fig. 6).

The cycloartenol synthase (*CAS*) expression increased by fivefold and 11-fold at 6 and 24 h after the onset of the high-temperature stress along with the application of EBR (4 μM) compared to the control, respectively (Fig. 6). At 6 and 24 h (without EBR application), the sterol methyltransferase (*SMT*) gene expression was enhanced compared to the control (Fig. 6d). The application of 4 μM of EBR at 6 h after the beginning of the high-temperature stress resulted in a fourfold increase in the expression of this gene in comparison with the control. However, applying 4 and 8 μM levels of EBR led to the expression of this gene to be increased 17-fold and 11-fold at 24 h after the beginning of the high-temperature stress, respectively (Fig. 6).

The sterol side chain reductase (*SSR*) expression was significantly enhanced (*p* < 0.01) under control (23 °C) at 6 (fourfold) and 24 h (3.5-fold) after the application of EBR (8 μM). The expression of this gene increased by 4.5-fold and sixfold under control at both 6 and 24 h after the application of 8 μM EBR, respectively (Fig. 6e). In addition, the high-temperature stress alone and in combination with the EBR application, significantly raised the expression of this gene compared to control (Fig. 6).

**Figure 7:**
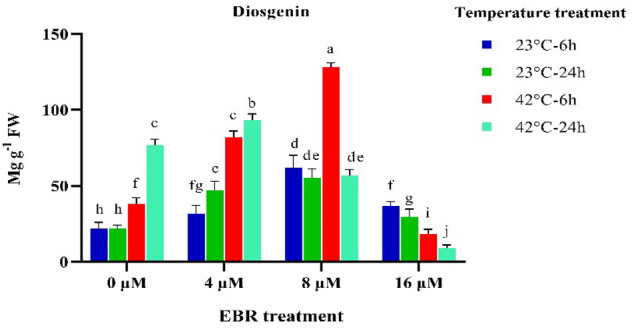
The effects of temperature, harvesting times, and different levels of EBR on diosgenin content*.* Duncan method at a 1% probability level was applied to compare mean values. The columns having similar letters had no significant difference.

The diosgenin content rose by twofold and 3.2-fold after 6 and 24 h from the beginning of high-temperature stress compared to the control (without the EBR application). Applying EBR under normal and high-temperature condition stimulated the expression of the genes closely associated with the biosynthesis of diosgenin, which in turn, raised the diosgenin content of fenugreek (Figs. 6 and 7). A sixfold rise in the content of diosgenin was observed following the application of 8 μM EBR under high-temperature (6 h) (Fig. 7).

## Discussion

In this study, the contents of chlorophyll and carotenoid content fell under high-temperature stress, probably due to the decreased synthesis and increased degradation of pigments (Fig. 1). One reason for the decrease in chlorophyll content is probably the increased activity of the chlorophyllase enzyme^27^. Furthermore, changes in the pathway of nitrogen metabolism due to the synthesis of compounds such as proline are the other important reasons^27,28^. Recent researches have shown that with increasing the stress intensity, the pigment content decreases^27,29^. Besides, the occurrence of carotenoid degradation may force the plants to develop some antioxidant pathways to cope with the imposed high-temperature stress^30^.

Applying EBR under normal temperature and high-temperature stress raised the chlorophyll and carotenoid contents (Fig. 1). The pigment contents were significantly increased with EBR application (4 and 8 μM) under normal temperature (23 °C) compared to the non-application of EBR. This result is consistent with the findings of other researchers^29^. It has been proven that the EBR improves the biosynthesis of these pigments^29^. BRs substantially reduce stress impacts on plants and raise nitrate reductase activity, which improves pigment content^22,31,32^. It was proven that EBR pre-treated plants showed significantly higher chlorophyll content and total protein content under both control and heat stress conditions compared to the control^33^. The elevated chlorophyll content following the application of EBR may be arisen out of the indirect impact of EBR on stimulating and protecting chlorophyll biosynthetic enzymes, activation of chlorophyll biosynthesis-related genes, including (*rca, rbcS* and *rbcl*), and affecting Rubisco activity under stress conditions^19,34^.

In the present study, temperature stress resulted in increased electrolyte leakage and MDA content; whereas, the application of EBR effectively reduced these two traits (Fig. 2). The use of different levels of EBR in plants grown under normal temperature condition also effectively led to the alleviation of the electrolyte leakage percentage and MDA content compared to the treatment of not using EBR. Previous studies have proven that the application of EBR leads to a decrease in MDA content in plants grown under normal condition^20,23^. The decline in the levels of MDA content suggests EBR mitigated the damaging effect of environmental stresses. There have been reports of the impact of brassinosteroids (BRs) on the reduction of MDA content and electrolyte leakage because of the protection of membrane lipids from the damage caused by reactive oxygen species^19,35^. It is worth mentioning that one of the impacts of EBR on plants would be the activation of the heat shock proteins that protect the biological molecules and cells against adverse conditions^22^.

The findings of this research indicated that the temperature stress and EBR raised the total soluble protein content compared to the normal temperature condition (Fig. 2). This enhancement might be attributed to the increased expression of some genes such as the ones related to primary metabolism, osmotic regulation, alteration of structure, and elimination of toxicity, as well as increased expression of late embryogenesis abundant proteins (LEA)^36^. In the current study, high-temperature stress and the EBR treatment lead to an increase in the activity of antioxidant enzymes.

It was demonstrated that using BRs induces antioxidant enzyme activity as well as non-enzymatic antioxidants. For instance, maize seedlings treated with brassinolide (BL) increased the activities of SOD, CAT, APX, carotenoid content, and ascorbic acid^37^. Antioxidant enzymes activity and mRNA expression of Cat A, Mn-SOD, Cat B, Cu/Zn-SOD, GR, and APX remarkably enhanced with EBL treatment under heavy metal stress in *Oryza sativa L*^38^. Increased expression of antioxidant enzymes as well as their activity after BRs application might occur due to increased DET gene expression, which improves plant tolerance to oxidative stress^35^. It was reported that the MDA content and the electrolyte leakage percentage in *Eucalyptus urophylla* rose in response to salt stress, while the application of EBR prevented any further increase in this traits^29^. It was emphasized that following the application of brassinolide and brassinazole, as an inhibitor of brassinolide synthesis, under low-temperature stress in *Medicago truncatula*, antioxidant enzymes activity increased and decreased, respectively^20^. EBR increases the plant tolerance to oxidative damage caused by ROS in response to environmental stimuli by adjusting the expression of genes directly or indirectly related to antioxidant enzymes^22,32^. Overall, findings from this study illustrate that high-temperature tolerance in fenugreek could be considerably improved after EBL application mainly through triggering ROS scavenging system (Fig. 3). In various researches, the positive and significant role of EBR on the quantity and quality of primary metabolites in plants has been proven^39,40^. In a research, the application of EBR on tomato plants under normal condition and cold stress led to a decrease in MDA content and an increase in the content of auxin, abscisic acid, and the activity of some antioxidant enzymes under normal and stress conditions. The findings of the present study are consistent with the results of recent research. Antioxidant enzymes activity and the proline content were enhanced by the 28-homobrassinolide treatment in the *Brassica juncea* under normal and cadmium stress^39^. The content of MDA under salinity stress in rice seedlings was reduced by EBL treatment^40^. Application of brassinosteroid in peppermint (*Mentha piperita* L.) under salinity hampered the death of the plant even at severe stress (150 mM) and prevented the negative impact of salinity stress by elevating the activities of antioxidant enzymes and reducing the lipid peroxidation^41^.

In the current study, high-temperature stress and EBR application both were followed by a significant increase in fenugreek H_2_O_2_ and NO contents (Fig. 4). Interestingly, the changes in fenugreek H_2_O_2_ and NO contents following the applied treatments were very similar, suggesting that these compounds play a prominent role under stress conditions. Noticeably, H_2_O_2_ and NO levels rose in both high-temperature-stressed and non-stressed (control) plants, suggesting that these compounds may be closely involved with BRs-mediated high-temperature tolerance. The results of the current study corroborate the previous findings which confirm the positive role of these compounds in mitigating environmental stresses and their interactions with EBR^19,31^. It is believed that EBR may raise plant H_2_O_2_ content under environmental stresses, thereby enhancing the production of NO^19,20,31^.

Various interactions between plant hormones induce a heterogeneous network of plant responses that make it challenging to predict plant performance in response to adverse conditions^42,43^. Moreover, brassinosteroid (BR) can regulate stress responses by cross-talking with other phytohormones^44,45^. In the current study, the content of abscisic acid rose significantly (*p* < 0.01) with the onset of high-temperature stress, and the use of exogenous EBR intensified this increase. Abscisic acid is known as a stress hormone that is influenced by stress and raises plant durability during abiotic stresses^46^. Moreover, ABA can decrease the damage of dehydration by closing the stomatal pore and maintaining the cellular water^47,48^. However, several antagonistic effects have been observed between signaling components of BRs and ABA under different stress conditions^46,49^. In addition, the endogenous content of auxin fell sharply with the onset of high-temperature stress, and the application of different levels of EBR largely prevented the reduction of this hormone. The exogenous EBR prohibited a significant decrease in auxin content, which in turn prevented a decline in plant growth under high-temperature stress (Fig. 5). The synergetic interactions are stated between BR and auxin in regulating the cellular processes related to growth, such as cell proliferation and cell expansion^50–52^. Furthermore, it was defined that BR and auxin are involved in several common cellular processes and BR can regulate cell elongation from auxin metabolism^53^. Findings from this study implied that the exogenous EBR probably increased the tolerance of fenugreek by activating the NO, H_2_O_2_, and ABA-dependent pathways^31^. In addition, these findings supported the existence of synergistic effects of EBR and auxin action, which has been confirmed by other researchers^31,32^. According to the findings of various experiments, it could be concluded that the interaction between EBR and abscisic acid is quite important in regulating a wide range of genes controlling photosynthesis, stomatal closure, synthesizing enzymatic and non-enzymatic antioxidants, and ultimately increasing plant tolerance^19,31,32^. Various studies on the role of plant hormones in response to adverse conditions have been performed, but the exact interaction between BR with auxin has not yet been determined, based on molecular information. The expression of many target genes that are involved in growth processes and stress response is commonly controlled by both BR and auxin^52,54,55^. Overall, the use of EBR treatment as a stimulant may induce some cellular signaling pathways associated with stress tolerance and reduce the adverse effects of stress on growth by increasing the content of growth-regulating hormones, such as ABA and auxin. Overall, we concluded that EBR diminishes the adverse effect of high-temperature stress by increasing the content of endogenous phytohormones, increasing the content of antioxidant enzymes, and controlling gene expression.

Being fed by the mevalonate pathway, biosynthesis of isoprenoids generally leads to the production of diosgenin. Squalene synthase (*SQS*) catalyzes the first enzymatic step of the isoprenoids pathway in the synthesis of sterols and triterpenes. In this current study, the expression of the studied genes (Fig. 6) and diosgenin content were influenced by high-temperature and the EBR treatments (Fig. 6). It was declared that the *SQS* gene expression rose under drought stress at different stages of *Glycyrrhiza glabra* growth^56^. In the present study, under control, EBR treatment (in the normal temperature) at all concentrations caused a significant decrease in the *SQS* gene expression (Fig. 4), whereas; This gene expression increased by sixfold and eightfold at 6 and 24 h after the onset of the high-temperature stress along with the application of EBR (8 μM) compared to the control, respectively (Fig. 6).

The *CAS* gene expression increased under high-temperature stress, especially at the more extended (24 h) (Fig. 6). EBR treatment under both short and long-term (6 and 24 h) high-temperature stresses significantly raised the expression of this gene in comparison with the control condition. Examining how cold plasma treatment influences the expression of *CAS* gene in fenugreek, it was revealed that the gene expression changes under various exposure times of this treatment^10^.

In the current research, the expression of the *SMT* gene was enhanced under high-temperature stress compared to the control, experiencing a severer rise when the stress duration elongated from 6 to 24 h. The results of this research showed that applying EBR under normal temperature reduced the expression of this gene. Under high-temperature stress along with 4 μM of EBR treatment, on the other hand, the *SMT* expression was significantly raised (Fig. 6). Interestingly, the use of different levels of EBR under control led to an increase in the *SSR* gene and a decrease in *SMT* expression; whereas, under high-temperature stress (especially 24 h), the biosynthetic pathway of diosgenin is driven the production of plant phytosterols by increasing the expression of *SMT* (the competitor gene in diosgenin synthetic pathway) to improve the plant's tolerance to high-temperature stress (Fig. 6). High-temperature stress has led to oxidative stress and increased the content of free radicals in the plant, and to overcome these conditions, the plant has diverted the biosynthetic pathway of diosgenin by raising *SMT* expression to the production of phytosterols. In 8 and 16 μM concentrations of EBR, the expression of this gene is probably reduced due to the plant's access to the exogenous application of EBR, and subsequently, the expression of the *SSR* gene increased. In this study, the expression of *SSR* after the application of EBR under high-temperature stress significantly fell compared to the control. Applying 8 μM of EBR significantly enhanced the expression of this gene, which means that, unlike the *SMT* gene, *SSR* responded only to the EBR treatment and not to the combination of EBR and high-temperature stress.

The current study demonstrated that the expression of the genes involved in the biosynthesis pathway of diosgenin and thus plant diosgenin content, considerably increased following the high-temperature stress without EBR application. When high-temperature stress (especially 6 h) was accompanied by different levels of EBR (especially 8 μM), a sixfold increase in diosgenin content compared to normal temperature (normal treatment without the application of EBR) was achieved (Fig. 7). Exposing the plants to the long-term temperature stress (24 h) led to a substantial decrease in diosgenin content in comparison with short-term stress (6 h). The EBR application and high-temperature (6 h) stimulated the expression of all of the investigated genes and raised the plant diosgenin content (Fig. 6). Applying EBR accompanied by high-temperature stress (6 h) raised the expression of both groups of partner (*SSR, CAS, SEP,* and *SQS*) and rival genes (*SMT*), which led to an increase in the plant diosgenin content. When it came to the long-term stress (24 h), the expression of the rival gene (*SMT*) rose more in comparison with other effective genes, which caused a fall in diosgenin content (Fig. 7).

The adverse environmental stimuli can cause diverse negative effects on plant performance as well as the quality and quantity of their products. Abiotic stresses effect in medicinal plants vary from those in other crops in that they may diminish biomass while increasing secondary metabolites. Therefore, the application of abiotic stresses in medicinal plants according to the purpose of their use (vegetative tissue or secondary metabolism) requires special expertise, particularly in identifying the intensity of this stress, and the plant growth stage as well as using various stimulants^26,57,58^. Numerous approaches have previously been utilized aimed at enhancing the secondary metabolites qualitatively and quantitatively in plants^1,10,56,59^. The conflicting results regarding how plant secondary metabolites contents vary following the application of different stimuli may highlight the prominent role of plant types in this matter. Depending on the factors like plant genotype, elicitor concentration and intensity and plant growth stage, identifying each of these factors could provide helpful information for increasing the content of plant secondary metabolites^1,10,56,59^. It was demonstrated that genes involved in the diosgenin biosynthesis pathway are sharply affected by melatonin application in plants grown under salinity^11^. For instance, the expression level of *CAS*, *SMT*, *SSR*, *SQS*, and *SEP* was significantly increased compared to normal condition. However, the expression pattern of these genes in the previous study is not completely consistent with the current research, which proves the necessity of investigating the effects of different stimuli, because the behavior of different genes under the influence of various stimuli is not the same. The findings of the current study revealed that EBR application (especially 8 µM), could induce the expression of some responsible for the diosgenin biosynthesis pathway as well as diosgenin content under high-temperature stress.

## Materials and methods

### Plant material and growth condition

The taxonomy of the studied plant was confirmed by a specialist botanist from the Ministry of Agriculture Jihad of Tehran, Iran. The plant material was obtained under the supervision and permission of the Ministry of Agriculture Jihad of Tehran, Iran as well as national guidelines, with all authors complying with all local and national guidelines. First, the seeds of fenugreek (Boshruyeh genotype) were sterilized with 1% (v/v) sodium hypochlorite solution for 10 min, then the seeds were rinsed several times with sterile water, and were planted in pots (35×30×25) including an equal ratio of coco peat, peat moss and sand. Five seeds were planted in each pot and incubated in a growth chamber where photoperiod, temperature, relative humidity, and light intensity were kept at 16/8 h (day: night), 22–25 °C, 60–65%, and 400 µmol m^−2^ s^−1^, respectively.

### EBR treatments

The fenugreek seedlings (in the 6-leaf stage) of different pots were divided into four groups after 4 weeks of planting^20^ and each one was sprayed with different concentrations of EBR (0, 4, 8, and 16 μM)^31,60,61^, and repeated after 6 h^23^. The stock solution of EBR (with the final concentration of 100 μM) (Sigma-Aldrich, USA) was prepared by dissolving the 4.8 mg of EBR in 3 mL of ethanol, in a 200-mL volumetric flask, and the final volume was adjusted to 100 mL using double distilled water. The required lower concentrations of EBR (2, 4, 6, and 16 μM) were prepared by diluting the stock solution. Surfactant (Tween 20; 0.1%) was added before the treatment. Each pot was sprayed with the same amount of EBR. This amount was 10 ml for each pot, so that the entire surface of the plant was completely wet. Solvent solutions at a concentration corresponding to the dilution of the reagents were used as controls in all experiments. Three biological replications were considered for each treatment. The EBR used in this study was purchased from Sigma-Merck Company (CAS number: 78821-43-9).

### High-temperature treatment

After EBR treatment, the treated plants were divided into two separate groups for high-temperature stress induction. The first group was incubated in the growth chamber as a control treatment at normal temperature (23 °C). The second group was transferred to another growth chamber as stress treatment experiencing 42 °C for 6, and 24 h individually^33^. Therefore, these investigation treatments included: concentrations of EBR (0, 4, 8, and 16 μM), different harvesting times (6, and 24 h) as well as temperatures (23 °C, and 42 °C). The leaves of each treatment were collected in 6, and 24 h after the onset of the high-temperature stress and kept at − 80 °C.

### Estimation of pigments

For determination of pigments, 0.25 g of grounded leaves sample was mixed with 10 ml of 80% acetone, and the samples were centrifuged at 3000 rpm for 30 min at 4 °C. Pigment contents were measured by a spectrophotometer (UV-1800; Shimadzu Corporation, Kyoto, Japan)^62^. The absorbance of samples was registered at 480, 649, and 665 nm and the traits were calculated based^63^ on the following formulas:

$${\text{Chlorophyll a}}\, = \,{12}.{\text{25A}}_{{{665}}} {-}{2}.{\text{79A}}_{{{649}}} ,$$

$${\text{Chlorophyll b}}\, = \,{21}.{\text{5A}}_{{{649}}} {-}{ 5}.{\text{1A}}_{{{665}}} ,$$

$${\text{Total carotenoids}}\, = \,\left( {{1}000{\text{A}}_{{{48}0}} {-}{1}.{\text{82Cha }}{-}{85}.0{\text{2Chb}}} \right)/{198}.$$

### Electrolyte leakage index (ELI)

200 mg of leaf samples were used for calculating the electrolyte leakage percent. The leaf samples of the same size (1×1 cm) were immersed in 10 ml of deionized water. A vacuum pump was used to absorb water entirely. The samples were shaken at 150 rpm for 45 min and a conductivity meter was used to measure the electrical conductivity (Weilheim, Germany). After the first measurement (L_0_), the falcons were incubated in boiling water (90 °C) for 10 min and shaken for 45 min (L_b_). Then, the electrolyte leakage percent was measured by the following formula^64^:

$$\% {\text{ ELI }} = \, \left( {{\text{L}}_{0} } \right)/\left( {{\text{L}}_{{\text{b}}} } \right) \, \times { 1}00.$$

### Lipid peroxidation assay

To measure the malondialdehyde (MDA) content, 250 mg of ground leaf tissue (in a mortar with liquid nitrogen) was extracted in 1% thiobarbituric acid solution (w/v) and centrifuged for 20 min at 3000 rpm. Next, a mixture composed of the supernatant (1 ml) and of thiobarbituric acid (2 ml; 0.5%) was created and heated at 90 °C for 40 min and afterward, the samples were incubated in an ice bath for cooling. After applying the centrifuge at 3000 rpm for 15 min, the absorbance at 532 and 600 nm was measured using a spectrophotometer (UV-1800; Shimadzu Corporation, Kyoto, Japan)^65^. Using the following equation, the quantity of malondialdehyde was calculated:

$${\text{MDA }} = \, \left[ {\left( {{\text{532 nm }}{-}{ 6}00{\text{ nm}}} \right)/\left( {{\text{QD }}\times{\text{ QF}}} \right)} \right] \, \times{\text{ DF}}. \, \,\,\,{\text{In this formula}},{\text{ QD }} = {\text{Cuvette diameter }}\left( {{\text{1cm}}} \right),{\text{ QF }} = {\text{ Extinction coefficient }}\left( {{\text{155 mmol}}/{\text{cm}}} \right),{\text{ and DF }} = {\text{ Dilution factor }}\left( {{2}0} \right).$$

### Total soluble protein and activity enzymes assay

0.5 g of the fenugreek leaves were crushed using liquid nitrogen and mixed with 50 mM of extraction phosphate buffer (pH = 7) at 4 °C. The homogenized samples were centrifuged at 3000 rpm for 30 min at 4 °C and the supernatant was used for total soluble protein and activity enzymes assay. A standard curve was developed using a series of Bovine Serum Albumin (BSA). 3 ml of Bradford solution was mixed with 100 μl of extract, and samples were vortexed to ensure mixing. The protein concentration of each sample was measured after 20 min using a spectrophotometer (UV-1800; Shimadzu Corporation, Kyoto, Japan) at 595 nm^66^. For CAT activity, 100 μL of the enzyme extract was added to 2.9 mL of reaction mixture containing 15 mM H_2_O_2_ and 50 mM phosphate buffer (pH 7). The degradation of H_2_O_2_ was measured by the decrease of absorbance at 240 nm for 1 min. One unit of CAT activity was defined as a decrease in absorbance at 240 nm of 0.01 per min^67^.

For APX activity, 100 μL of the enzyme extract was added to 2.9 mL of reaction mixture containing 50 mM phosphate buffer (pH 7), 0.5 mM ascorbic acid, and 1 mM H_2_O_2_. The decrease of absorbance at 290 nm during 1 min was measured. One unit of APX activity was defined as the enzyme that oxidizes 1 μmol of ascorbate per min^68^. For SOD activity, 100 μL of enzyme extract was added to 2.9 mL of reaction mixture containing 50 mM phosphate buffer (pH 7), 5 mM methionine, 100 μM EDTA, 65 μM NBT, and 40 μL of 0.15 mM riboflavin. The tubes were then placed in a fluorescent light incubator (40 W, 10 min), and the formation of blue formazan was monitored by recording the absorbance at 560 nm. One unit of SOD activity is defined as the enzyme that causes a 50% inhibition of NBT reduction under assay conditions^69^.

GP_X_ activity was measured according to the protocol suggested using a spectrophotometer (UV-1800; Shimadzu Corporation, Kyoto, Japan) at 340 nm. The materials for this measurement included 2200 μl phosphate buffer (pH 7) 50 mM, 50 μl regenerated glutathione 10 mM, 100 μl Na_2_EDTA 1 mM, 100 μl NADPH 1 mM, distilled water 480 μl, and enzyme extract 100 μl^70^. For GR activity based on the decrease in absorbance at 340 nm due to NADPH oxidation, 100 μL of enzyme extract was added to a reaction mixture containing 1.5 mL of 50 mM phosphate buffer (pH 7), 150 μL of 20 mM GSSG, 1 mL of distilled water and 150 μL of 2 mM NADPH (dissolved in Tris–HCl buffer, pH 7), in a final volume of 3.0 mL^71^.

### Measurement of H_2_O_2_ and NO contents

First, five ml of 1% trichloroacetic acid and 0.2 g charcoal were added to 0.5 g of powdered leaf tissue. After being centrifuged at 10,000 rpm for 15 min, the mixture was filtered. Then, the prepared solutions (1 ml) were combined with 1 ml of colorimetric reagent. Meanwhile, 8 mg of catalase was introduced to these solutions and then they were incubated at 25 °C for 10 min^72^. Finally, absorbance was recorded at 390 nm by a spectrophotometer (Shimadzu Corporation, Japan). Finally, H_2_O_2_ content was plotted and measured using standard (Sigma, USA, CAS Number: 7722-84-1) curves. Furthermore, in order to determine the nitric oxide content, 3 ml of 50 mM acetic acid was added to the powdered leaves (0.6 g)^73^. After centrifuging the solution at 10,000 rpm for 20 min at 4 °C, the supernatant was gathered. The extraction buffer (1 ml) was used to wash the pellet and then, vortex and filtration were carried out. Lastly, 1 ml of Griess reagent was blended with 1 ml of filtrate and the mixture was kept at 4 °C for 30 min. NO content was determined with the NaNO_2_ standard curve (Sigma, USA, CAS Number: 7632-00-0, EC Number: 231-555-9). By measuring the absorbance at a wavelength of 540 nm and comparing the obtained graph with the standard one provided for NaNO_2_, NO content was determined.

### Auxin content

For this purpose, 1.5 g of powdered leaves were thoroughly homogenized in 20 ml of the water–methanol mixture in equal proportion. After 15 min of centrifugation at 3000 rpm, the solution was put in a freeze dryer to be chilled and evaporated, and one milliliter of 80% methanol was added. Before HPLC analysis (Agilent Technologies Inc., USA, 1200 series), the reconstituted eluate was filtered through a 0.45 m Whatman glass microfiber filter to remove impurities. The HPLC column was heated to 30 ^◦^C with a 0.45% formic acid: acetonitrile gradient (0–5 min, 95:5% (v/v); 5–6 min, 95:5% to 0:100% (v/v); 6–16 min, 0:100% (v/v))^74^. Eventually, the auxin concentration in leaf tissue was determined at the flow rate of 0.8 ml/min using an HPLC column (C18, 4.6 µm, 250 mm length, 5 mm diameter)^75^. The volume injection and retention were 20 µl and approximately 9 min, respectively. Peak spiking, retention time, and spectral properties were applied to identify the peak position. Furthermore, a linear regression equation of standard (Sigma, USA, CAS Number: 87-51-4, EC Number: 201-748-2) calibration curves was applied to determine the auxin concentration. The correlation coefficient (R^2^) was calculated at 0.99 for the six different concentrations that were evaluated. The target component was quantified by the peak areas at the maximum wavelength of 260 nm.

### Content of abscisic acid

First, 2.0 g of fresh leaves were well-ground and subjected to 1 ml of a solution composed of methanol, ethyl acetate, and acetic acid at the rate of 1:50:49. Then, 20 mg of butylated hydroxytoluene (BTH) was introduced to this solution as antioxidant^26^. The solution was then passed via a 0.45 µm Whatman glass microfiber filter and by adding distilled water, the final volume was raised to 100 ml. Under vacuum and at a temperature of 35 °C, a rotary apparatus was applied to evaporate the solvent^76^. Then, the samples were exposed to 10 ml of 50 mM hydrogen phosphate (pH = 7) followed by filtration. After adjusting the pH to 4.2^75^. 10 ml of ethyl acetate was added to the solution. The solution was then treated with 0.1 g of sodium sulfate to dehydrate the abscisic acid-containing ethyl acetate solution. After adding 5 ml of methyl chloride to the dried extract to evaporate it, the solutions were incubated at room temperature for 24 h. Finally, the extract was combined with 3 ml of a 1% acetic acid-containing methanol solution and filtered.

A HPLC column (Agilent Technologies Inc., USA, 1200 series, C18, 4.2 µm, 250 mm length, and 5 mm diameter) was applied with an interval of 4 to 5 min based on standard abscisic acid to determine the concentration of this important compound. In the chromatographic separation stage, two mobile phases, including a medium comprised of water/ acetonitrile/formic (A) acid in the volume ratio of 94.9:5:0.1, respectively, and another medium comprised of the same materials in the volume ratio of 10:89.9:0.1 (B), respectively, were applied. The elution program maintained 100% A for 5 min, followed by two consecutive linear gradients from 0 to 6% B in 10 min, and from 6 to 100% B in 5 min, and finally, 100% B was maintained for another 5 min^75^.

A flow rate of 0.8 ml/min was applied at a wavelength of 254 nm. The volume injection and retention were 20 µl and approximately 11 min, respectively. Finally, quantification was carried out given the specific area of the peak in relation to the retention time of the standard abscisic acid sample. ABA standard was obtained from Sigma, USA (CAS Number: 21293-29-8, EC Number: 244-319-5). The correlation coefficient (R^2^) was calculated at 0.98 for the six different concentrations that were evaluated. The target component was quantified by the peak areas at the maximum wavelength of 260 nm.

### Gene expression analysis

By applying the RNeasy plant mini kit (Qiagen) based on the instruction of the manufacturer, total RNA was extracted. The quality and quantity of RNA were determined using 1% agarose gel and a NanoDrop spectrophotometer. Also, the DNA contamination was resolved using the DNA-free kit (Qiagen) and the cDNA synthesized with the iScript cDNA synthesis kit (Bio-Rad, CA, USA). One µl of the synthesized cDNA was used at the 10 µl PCR reaction by applying an Amplicon EvaGreen Kit. The qRT-PCR reactions included 35 cycles of 95 °C for 20 s and 61 °C for 40 s preceded by 95 °C for 10 min. Table 1 represents the primers used in the current study. Furthermore, to ensure that the samples were uncontaminated, a negative control was used during the experiment. The primers were designed by applying the Primer 3 Plus online program (https://www.bioinformatics.nl/cgi-bin/primer3plus/primer3plus.cgi), and their accuracy was checked using Oligo Analyzer v.3.1 (http://eu.idtdna.com/calc/analyzer). The gene expression analysis was conducted in three biological and technical replications. The fenugreek *GAPDH* gene was used for initially normalizing the relative expression of genes.

**Table 1:** Primers used in this study and their characteristics.

Primer name	(5′–3′) primer sequence		Accession number	Length (bp)	Annealing temperature
	Forward	Reverse			
SSR	AGGTGGGAGATATGCTAGAATGGG	CATTCTGTGTGTCTCCCTGCC	MK060115.1	152	61
SMT	GAGAAGGCTGCAGAGGGTCTAG	CACAGTAACTCATGACAACAGCAACCT	MK060114.1	140	62
SEP	CTATTCTCAGAAGGACCGATCTC	CAGATGCACCAGAGAGTAATCG	KX14846	168	60
CAS	AAGAGAGATCCAACACCACTGC	TACATGACGACGGTATTCTCCC	KX148475.1	17	60
SQS	GGCTCAACCATGATTCTCATACTG	TACCCACTGTTCCATTGCTATCC	KX148475.1	133	61
GAPDH (reference gene)	ATGTTAAATGATGCAGCCCTTCCACCTCTC	TATGTTTGTTGTTGGTGTCAACGAGCAACGAATACAAG	XM_010679634.2	230	61

### Evaluation of diosgenin content

First, 1 g of powdered plant leaves was dissolved in 20 ml of 96% ethanol, and the mixture was thoroughly homogenized. Next, the solutions were ultrasonicated for 30 min and then, treated with 20 ml of sulfuric acid (2N), and kept at 95 °C for 2 h. Separating the solution ingredients by pure n-hexane was carried out three times. It aimed at neutralizing the acidic state, the solution was well-washed with sodium hydroxide (1 M). The resulting solution was then washed with distilled water twice. Afterward, the n-hexane solution containing diosgenin was dried by subjecting it to a vacuum generated by a rotating equipment, and then dehydrated with 0.2 g of anhydrous sodium sulfate. After dissolving the obtained extract in acetonitrile, it was passed through a 0.22 μm Whatman glass microfiber filter^58^. The experimental conditions were the isocratic binary system of acetonitrile: water (90:10). The process of determining the diosgenin content was carried out by utilizing a HPLC column (Agilent Technologies Inc., USA, 1200 series, C18, 4.2 µm, 250 mm length, 5 mm diameter) at the flow rate of 0.8 ml/min and the wavelength of 210 nm. Different concentrations of diosgenin (Sigma-Aldrich-Germany; CAS Number: 512049) including, 100, 150, 300, 500, and 1000 ppm were produced by dissolving in acetonitrile before placing the samples in ultrasonic for 30 min^77^. The volume injection and retention were 30 µl and approximately 23 min, respectively. Finally, the correlation coefficient (R^2^) was calculated at 0.99 for the data sets.

### Statistical analysis

The differences among the evaluated traits were assessed in three replicates applying one-way analysis of variance (ANOVA) in SPSS software 19 (SPSS Inc., Chicago, IL, USA, 1998). According to the treatments applied, this study was conducted as a factorial in a completely randomized design with three replications. Furthermore, the means comparison was done by Duncan’s test at a 1% level. Furthermore, melting and amplification curve analyses were accurately carried out to confirm the accuracy of the data before analyzing the expression data of the genes. To analyze the data obtained from real-time PCR, the relative expression of each gene was measured according to the relative standard curve method based on the formula 2^−ΔΔCt^^78^.

## Data Availability

The datasets used and/or analyzed during the current study are available from the corresponding author on reasonable request.
